# AMPLIFYING COMMUNITY ENGAGEMENT TO INCREASE REPRESENTATION AND APPLICABILITY OF CAREGIVING RESEARCH

**DOI:** 10.1093/geroni/igac059.325

**Published:** 2022-12-20

**Authors:** Ishan Williams, Randy Jones, Travonia Brown-Hughes

**Affiliations:** University of Virginia, School of Nursing, Charlottesville, Virginia, United States; University of Virginia School of Nursing, Charottesville, Virginia, United States; Hampton University, Hampton, Virginia, United States

## Abstract

Alzheimer’s disease and related diseases (ADRD) disproportionately affect persons of African American ethnicity, yet persons who identify as Black/African American are consistently and markedly underrepresented in Alzheimer’s research. Prior research suggests that a complex array of factors, from mistrust in medical research to non-inclusive recruitment approaches, have led to the disparity. With the growing rates of ADRD among racial/ethnic groups in the US, it is imperative that research scientists develop interventions and clinical research that are culturally informed and meaningful to the lives of diverse caregivers. The goal of our research is to demonstrate the importance of community engagement and culturally informed interventions, and to offer best practices to advance the science of caregiver recruitment, which may ultimately improve overall representation across racial/ethnic caregiver groups. Research findings will highlight the variety of recruitment strategies used to build trust and more sustainable relationships with diverse communities often underrepresented in research.

